# CMTS-GNN: a cross-modal temporal-spectral graph neural network with cognitive network explainability

**DOI:** 10.3389/fneur.2025.1700161

**Published:** 2025-10-30

**Authors:** Yi Wang, Lu Meng, Yuying Fan

**Affiliations:** ^1^School of Information Science and Engineering, Northeastern University, Shenyang, China; ^2^Department of Pediatrics, Shengjing Hospital of China Medical University, Shenyang, China

**Keywords:** infantile spasms, cognitive control, explainability analysis, cross-modal, brain regions

## Abstract

Infantile spasms (IS) represent a severe form of epileptic encephalopathy occurring in early infancy. Timely and accurate detection is critical, as delays or misdiagnosis are associated with adverse neurodevelopmental outcomes that can impair perceptual, cognitive, and affective development. Conventional EEG analysis is often challenged by the complexity, heterogeneity, and large volume of IS data, rendering manual review both time-intensive and susceptible to inter-rater variability. To address these challenges, we introduce CMTS-GNN—a Cross-Modal Temporal—Spectral Graph Neural Network. This model integrates complementary information from temporal and spectral EEG representations through bidirectional cross-modal attention and gated fusion mechanisms. It further incorporates explicit modeling of brain-region connectivity to capture functional interactions that underlie perceptual processing, cognitive control, and affective dynamics. By doing so, CMTS-GNN aims to improve both detection accuracy and interpretability. We evaluated the proposed model on an in-house infantile spasms dataset and the publicly available CHB-MIT epilepsy dataset. Evaluation protocols included five-fold cross-validation and subject-independent schemes (leave-one-subject-out/leave-one-patient-out). On our in-house dataset, five-fold cross-validation resulted in an accuracy of 99.02%, precision of 98.96%, recall of 97.47%, F1-score of 98.20%, and AUC of 99.27%. For the CHB-MIT dataset, the same protocol yielded an accuracy of 98.54%, precision of 98.31%, recall of 98.71%, F1-score of 98.47%, and AUC of 98.87, outperforming several recent approaches across most metrics. Subject-independent evaluations further confirmed the model's robustness and generalizability across different patients. Importantly, by modeling connectivity across brain regions, CMTS-GNN provides clinically meaningful explanations for its decisions, enhancing interpretability. In summary, CMTS-GNN offers an accurate, generalizable, and interpretable framework for automated IS detection from EEG. It holds potential to support earlier clinical intervention, thereby helping to mitigate long-term perceptual, cognitive, and affective morbidity in affected infants.

## 1 Introduction

Infantile spasms (IS) represent a severe form of epileptic encephalopathy occurring in early infancy, characterized by stereotypical epileptic spasms, a highly disorganized electroencephalographic pattern known as hypsarrhythmia, and developmental stagnation or regression that may compromise perception, cognition, and affective development (1). IS is widely classified within the spectrum of West syndrome and exhibits marked clinical heterogeneity (2, 3). The global incidence is approximately 0.02% to 0.05%, with no significant sex differences. Most cases present between 4 and 9 months of age, with a peak onset around 6 months (4, 5). This developmental window coincides with critical periods for sensory–perceptual integration and early cognitive–affective maturation. The typical spasms present as brief, repetitive clusters, characterized by flexion or extension of the trunk, often accompanied by autonomic symptoms such as ocular deviation and alterations in respiratory rhythm. These events are more frequent or pronounced during wakefulness or transitional sleep states (6, 7). Due to the presence of atypical spasm manifestations in some cases, IS can be easily misdiagnosed as other infantile movement disorders, leading to delayed diagnosis and potentially irreversible neurodevelopmental impairment that affects perceptual, cognitive, and affective trajectories (8).

Electroencephalography (EEG) is a non-invasive technique that records neuronal electrophysiological activity via scalp electrodes. It serves as a critical tool in the clinical diagnosis of epilepsy and related encephalopathies, offering high temporal resolution and cost-effectiveness (9). In infantile spasms, EEG holds central diagnostic value. During ictal episodes, characteristic changes such as voltage attenuation and bursts of fast rhythms can be observed (10). Current clinical diagnosis relies on prolonged, synchronized video-EEG monitoring, which requires manual interpretation by trained clinicians to detect ictal events and abnormal discharge patterns (11). However, this approach faces three major challenges. First, EEG patterns associated with infantile spasms are highly heterogeneous–not only do they vary significantly between individuals, but they also exhibit dynamic fluctuations over time within the same patient, reflecting complex spatiotemporal variability in epileptic discharges (12). Second, prolonged monitoring generates a large volume of data, making manual analysis time-consuming and labor-intensive, which results in low diagnostic efficiency (13). Third, EEG interpretation is highly dependent on clinician expertise, and inter-rater consistency among experts is limited, which hinders the standardization of diagnosis and treatment (14, 15). These challenges highlight the urgent need for automated detection technologies in the diagnosis and management of infantile spasms.

In recent years, deep learning-based end-to-end models have shown promising performance in the detection of epileptiform discharges, offering a feasible pathway for the automated recognition of EEG signals (16–18). Zhou et al. (19) developed a convolutional neural network (CNN) framework for automatic seizure detection, which processes raw EEG signals directly in the frequency domain without the need for manual feature extraction. Cao et al. (20) proposed a deep transfer learning-based feature fusion algorithm for multi-state epileptic EEG classification. The method constructs sub-band mean amplitude spectrum maps to characterize brain rhythm activity and leverages five ImageNet-pretrained deep neural networks (AlexNet, VGG19, Inception-v3, ResNet152, and Inception-ResNet-v2) to extract and fuse discriminative EEG features. In the study by Tsiouris et al. (21), a long short-term memory (LSTM) network was employed to extract temporal information from EEG segments for seizure detection. Further advancing this approach, Yao et al. (22) integrated an attention mechanism into the LSTM framework to enhance the model's ability to detect epileptic seizure. Recent studies have revealed specific patterns of correlation among neural signals originating from distinct brain regions (23). Brain networks are typically modeled as graph structures due to their inherently non-Euclidean nature. While traditional convolutional neural networks (CNNs) are well-suited for processing regular, Euclidean data such as images, they are limited in capturing the complex topological properties of brain connectivity. To more effectively leverage the spatial and structural information embedded in brain networks, graph neural networks (GNNs) have been introduced (24). Recent advances have adopted graph convolutional approaches, modeling EEG electrode channels as nodes within a topological graph, where edges denote functional or anatomical connections between electrodes. This framework mitigates the constraints imposed by fixed convolutional kernels in conventional CNNs and enables the retention of more intricate structural characteristics embedded in EEG data (25–27). Meng et al. (14) proposed a method based on Graph Convolutional Networks (GCNs) to automatically identify Electrical Status Epilepticus during Sleep (ESES) from electroencephalogram (EEG) recordings. Their model preserves the intrinsic graph structure of EEG signals and leverages both time-domain and frequency-domain features, achieving higher accuracy and generalizability compared to traditional approaches such as template matching and conventional machine learning models. However, this method has certain limitations, particularly when dealing with dynamic temporal data. EEG signals exhibit not only spatial correlations across electrodes but also strong temporal dependencies. In conventional graph classification tasks, the topological structure and temporal dynamics of the graph may not be fully exploited simultaneously. In the context of EEG, the signal at each electrode is not only correlated with signals from other electrodes but also shows a clear dependency over time. If a GNN fails to account for this temporal dependency, critical information may be lost, potentially degrading classification performance.

Most existing methods primarily focus on a single modality, with limited consideration of the relationships among temporal, spatial, and frequency domains. Although current deep learning approaches are capable of capturing temporal dependencies, they often lack explicit modeling of interactions across different modalities. Our work addresses this limitation by introducing a multimodal attention mechanism to explicitly model the dependencies between temporal and frequency features, thereby bridging this gap. In addition, most previous studies only validated their models on a single dataset, raising concerns about generalizability. Furthermore, existing explainability analyses have mainly targeted adult epilepsy datasets, whereas our study systematically analyzes explainability specifically on infantile spasm datasets, providing valuable references for clinical translation. To address these challenges, we propose a novel Cross-Modal Temporal-Spectral Graph Neural Network (CMTS-GNN) that integrates both temporal and spectral information for spasm detection. The proposed model combines multi-scale temporal feature extraction, spectral-domain modeling, and a cross-modal attention mechanism to fully leverage the temporal, frequency, and spatial characteristics of EEG data. CMTS-GNN has been evaluated on both a proprietary dataset and a public benchmark dataset to validate its generalization ability. We employ five-fold cross-validation for comprehensive performance assessment and conduct independent validation to ensure complete separation of patient data between the training and test sets, thereby preventing data leakage and overfitting.The main contributions of this work are summarized as follows:

- We proposed CMTS-GNN, a cross-modal temporal-spectral graph neural network that integrates temporal and spectral EEG features via bidirectional attention and gated fusion, enabling comprehensive and robust modeling of spatio-temporal patterns for infant spasms detection.
- The model explicitly divides EEG channels into five regions—frontal, central, parietal, occipital, and temporal lobes—based on the international 10–20 electrode system. Region-wise attention pooling is then employed to adaptively aggregate salient features within each brain region. This region-aware design significantly enhances the model's spatial specificity and interpretability in representing brain functional areas. Using attribution methods, we spatially visualize the basis of the model's decisions and observe that its focus closely aligns with the clinically recognized epileptogenic zones of infantile spasms. This further strengthens the model's explainability and medical credibility, laying a solid foundation for future clinical translation.
- The proposed model not only achieves state-of-the-art accuracy and robustness on the dedicated infantile spasm dataset but also demonstrates strong generalization performance in cross-domain transfer experiments on the public CHB-MIT epilepsy dataset. These results suggest that the framework presented in this study can efficiently detect infantile spasms as well as effectively recognize epileptic seizures, highlighting its significant potential for widespread clinical application.

The remainder of this paper is organized as follows. Section 2 provides a detailed description of the methods used in this study. Our experimental results are presented in Section 3. Finally, Section 4 concludes the study.

## 2 Materials and methods

### 2.1 Datasets

Datasets A. We evaluated the proposed method on two electroencephalogram (EEG) datasets. Dataset A was obtained from Shengjing Hospital of China Medical University and contains EEG recordings from 40 pediatric patients diagnosed with infantile spasms. All participants were younger than two years, and electrodes were positioned following the international 10-20 system. The cohort comprises 16 females and 24 males. Table 1 summarizes patient-level demographics and recording information.

**Table 1:** Infantile spasms A dataset: participant demographics and recording summary.

**Participants 01–20**						**Participants 21–40**					
**ID**	**Gender**	**Age**	**Spasms (n)**	**EEG (h)**	**Non-spasm**	**ID**	**Gender**	**Age**	**Spasms (n)**	**EEG (h)**	**Non-spasm**
01	M	1y1m	8	5.5	42	21	M	10m	32	12	92
02	F	4m	6	3.5	27	22	F	7m	8	3.5	27
03	F	8m	9	6	46	23	F	4m	20	6.5	50
04	M	5m	21	9	69	24	F	1y5m	13	4	31
05	M	3m	6	3	23	25	M	1y7m	23	7	53
06	M	6m	10	3	23	26	F	6m	12	5.5	42
07	M	1y8m	17	4.5	34	27	M	5m	19	8	61
08	M	5m	9	3.5	27	28	M	5m	9	3	23
09	M	1y	15	4	31	29	M	4m	24	7.5	57
10	F	7m	13	4	31	30	M	5m	27	9	69
11	M	3m	10	3	23	31	M	1y3m	22	6	46
12	F	4.5m	13	4	31	32	F	53 d	11	4	31
13	M	7m	14	4.5	34	33	M	6m	12	4.5	34
14	M	1y7m	9	3	23	34	F	10m	16	5	38
15	F	2m	11	3	23	35	F	7m	17	5	38
16	F	3m	15	4.5	34	36	M	6m	17	6	46
17	F	4m	18	7	53	37	M	9m	18	5.5	42
18	M	1y8m	14	6	46	38	F	10m	42	12	92
19	M	3m	17	8	61	39	F	4m	37	12.5	96
20	M	6m	13	6.5	50	40	M	9m	33	12	92

Age reported as years (y), months (m), or days (d). Gender: M (male), F (female).

Dataset B (CHB-MIT). The CHB-MIT dataset (28) used in this study was collected at Boston Children's Hospital and consists of pediatric EEG from children with epilepsy. Signals were recorded with 23 scalp electrodes arranged according to the 10-20 standard, yielding 844 hours of continuous EEG. The database contains 198 seizure events. Recordings are available for 24 subjects in total, but patient 24 was excluded here because detailed metadata and channel information are missing for that subject, which was added in a later phase of collection. All EEG was sampled at 256 Hz, and seizure onset/offset times were manually annotated. Most recording files are about one hour in duration, although some for particular patients extend to two or four hours.

### 2.2 Data processing

Due to variations in the number of recording channels and sampling frequencies across datasets, a standardized preprocessing pipeline was applied. Specifically, 16 commonly used EEG channels (Fp1, Fp2, F3, F4, C3, C4, P3, P4, O1, O2, F7, F8, T3, T4, T5, T6) were selected, and all signals were resampled to a uniform frequency of 250 Hz. The EEG recordings for each patient were then segmented into 5-second epochs, and each segment was labeled by experienced neurologists. Given that EEG signals are often contaminated by power line interference, electromyographic (EMG) artifacts, and ocular movements during acquisition, a multi-stage filtering strategy was adopted for signal denoising. A bandpass filter ranging from 0.7 to 40 Hz was applied to suppress both power line noise and high-frequency artifacts, while preserving seizure-related features and minimizing information loss. This approach helps prevent the loss of critical ictal waveforms due to over-filtering. To address inter-subject variability in signal amplitude, a dynamic gain control mechanism was introduced. Specifically, an average reference was applied during the preprocessing stage to reduce common-mode interference. Subsequently, Z-score normalization was performed on each channel, ensuring that the mean and variance of the signals were standardized to zero and one, respectively. This normalization strategy not only improves model convergence during training but also enhances its generalization ability across heterogeneous datasets.

### 2.3 Temporal graph construction

To simultaneously capture temporal dynamics and inter-channel dependencies within the temporal branch of CMTS-GNN, each 5-s EEG segment is represented as a temporal graph. We consider segments with *C* = 16 channels and *T* sampling points per segment. In this graph, nodes correspond to electrode channels; node features are the standardized time series of each channel; and edge weights quantify the strength of time-varying functional connectivity. The total number of nodes is 16.

Let the raw EEG matrix be **X** ∈ ℝ^*C*×*T*^. We apply per-channel *z*-score standardization to obtain **Z**:


(1) $$\begin{array}{c} Z_{i,t}=\frac{X_{i,t}-\mu_{i}}{\sigma_{i}+\varepsilon }, \end{array}$$

where *X*_*i,t*_ is the amplitude of channel *i* at time *t*; *Z*_*i,t*_ is the standardized amplitude; μ_*i*_ and σ_*i*_ denote the mean and standard deviation of channel *i*; and ε > 0 is a stability constant. The vector **z**_*i*_ = (*Z*_*i*,1_, …, *Z*_*i,T*_) serves as the feature of node *i*.

To characterize time-varying inter-channel relations, we compute sliding-window Pearson correlations over **Z**. With window length *L* and step size *S*, the number of windows is *K* = ⌊(*T* − *L*)/*S*⌋ + 1. For the *k*-th window, let ${\text{z}}_{i}^{\left(k\right)}$ and ${\text{z}}_{j}^{\left(k\right)}$ denote the length-*L* subsequences of channels *i* and *j*. Their correlation is


(2) $$\begin{array}{c} r_{ij}^{\left(k\right)}=\frac{\mathrm{cov}({\text{z}}_{i}^{\left(k\right)},{\text{z}}_{j}^{\left(k\right)})}{\sigma_{i}^{\left(k\right)}\sigma_{j}^{\left(k\right)}+\varepsilon }, \end{array}$$

where cov(·, ·) is the sample covariance and $\sigma_{i}^{\left(k\right)},\sigma_{j}^{\left(k\right)}$ are the sample standard deviations of the corresponding subsequences.

Based on these dynamic correlations, we construct a *fully connected, undirected, weighted graph without self-loops* 𝒢 = (𝒱, ℰ, **W**). For each unordered pair {*i, j*} with *i* ≠ *j*, the edge weight is defined as the average correlation across windows:


(3) $$\begin{array}{c} w_{ij}=\frac{1}{K}\overset{K}{\underset{k=1}{\sum }}r_{ij}^{\left(k\right)}\text{ with }w_{ij}=w_{ji},w_{ii}=0. \end{array}$$

Equivalently, ℰ = {{*i, j*} ∣ 1 ≤ *i* < *j* ≤ *C*} and the adjacency (weight) matrix **W** = [*w*_*ij*_] is symmetric.

Through this construction, the graph topology explicitly encodes cross-channel functional connectivity, while the node features preserve complete time-domain information. This representation enables CMTS-GNN to exploit complementary temporal and spatial cues in subsequent processing.

### 2.4 Spectral graph construction

The proposed CMTS-GNN integrates temporal and frequency-domain information within a unified graph-based framework to comprehensively capture the temporal dynamics, spectral characteristics, and spatial dependencies of infantile spasms (IS) EEG signals. In the temporal branch, the raw time series of each EEG channel ${\text{x}}_{i}\in {\mathbb{R}}^{T}$ is processed by a multi-scale encoder composed of three parallel one-dimensional convolutional branches with kernel sizes *k* ∈ {100, 50, 25}:


(4) $$\begin{array}{c} {\text{h}}_{i}^{\left(k\right)}=\text{ReLU}\left(\text{BN}\left({\text{x}}_{i}*{\text{W}}_{k}\right)\right), \end{array}$$

where **W**_*k*_ is the convolution kernel for scale *k*, BN(·) denotes batch normalization, and * represents the one-dimensional convolution operator. The outputs from all scales are concatenated along the channel dimension and passed through global average pooling to produce compact multi-scale temporal features:


(5) $$\begin{array}{c} {\text{h}}_{i}^{\text{temp}}=\text{GAP}\left({\|}_{k}{\text{h}}_{i}^{\left(k\right)}\right). \end{array}$$

Both the temporal graph, constructed from dynamic functional connectivity, and the frequency-domain graph, constructed from the weighted phase lag index (wPLI), are processed using edge-conditioned graph convolution, in which edge attributes are transformed into learnable kernels for message passing:


(6) $$\begin{array}{c} {\text{h}}_{i}^{\prime }=\sigma \left(\frac{1}{\left|N\left(i\right)\right|}\underset{j\in N\left(i\right)}{\sum }{\text{W}}_{\phi \left({\text{e}}_{ij}\right)}{\text{h}}_{j}\right), \end{array}$$

where 𝒩(*i*) denotes the neighbor set of node *i*, **e**_*ij*_ is the edge attribute (either a DFC or wPLI weight), ϕ(·) is an MLP that maps edge attributes to convolution kernels, and σ is the ReLU activation.

Anatomical priors are incorporated by grouping EEG channels into *R* = 5 brain regions ${\left\{V_{r}\right\}}_{r=1}^{R}$ (frontal, central, parietal, occipital, and temporal). Within each region, features are aggregated via attention pooling:


(7) $$\begin{array}{c} {\text{g}}_{r}=\underset{i\in V_{r}}{\sum }\alpha_{i}^{\left(r\right)}{\text{h}}_{i},\;\alpha_{i}^{\left(r\right)}=\frac{\exp\left({\text{w}}_{r}^{⊤}{\text{h}}_{i}\right)}{\sum_{j\in V_{r}}\exp\left({\text{w}}_{r}^{⊤}{\text{h}}_{j}\right)}, \end{array}$$

where **w**_*r*_ is a learnable vector for region *r*.

Cross-modal interaction is enabled by a bidirectional multi-head attention mechanism, allowing temporal features to attend to spectral features and vice versa, based on the scaled dot-product attention:


(8) $$\begin{array}{c} \text{Attention}\left(\text{Q},\text{K},\text{V}\right)=\text{softmax}\left(\frac{\text{Q}{\text{K}}^{⊤}}{\sqrt{d_{h}}}\right)\text{V}, \end{array}$$

where *d*_*h*_ is the per-head dimensionality.

The raw and cross-enhanced features are then fused through a gated mechanism:


(9) $$\begin{array}{c} \text{u}=\sigma \left({\text{W}}_{g}\left[{\text{h}}^{\text{raw}};{\text{h}}^{\text{enh}}\right]\right),\;{\text{h}}^{\text{fused}}=\text{u}⊙{\text{h}}^{\text{enh}}+\left(1-\text{u}\right)⊙{\text{h}}^{\text{raw}}, \end{array}$$

where ⊙ denotes element-wise multiplication and σ is the sigmoid function.

Finally, the fused temporal and spectral regional features are concatenated, flattened, and passed through a fully connected classifier to produce the final prediction. This end-to-end architecture allows CMTS-GNN to jointly exploit temporal, spectral, and spatial cues for robust automated detection of infantile spasms.

### 2.5 CMTS-GNN overview

**Figure 1:**
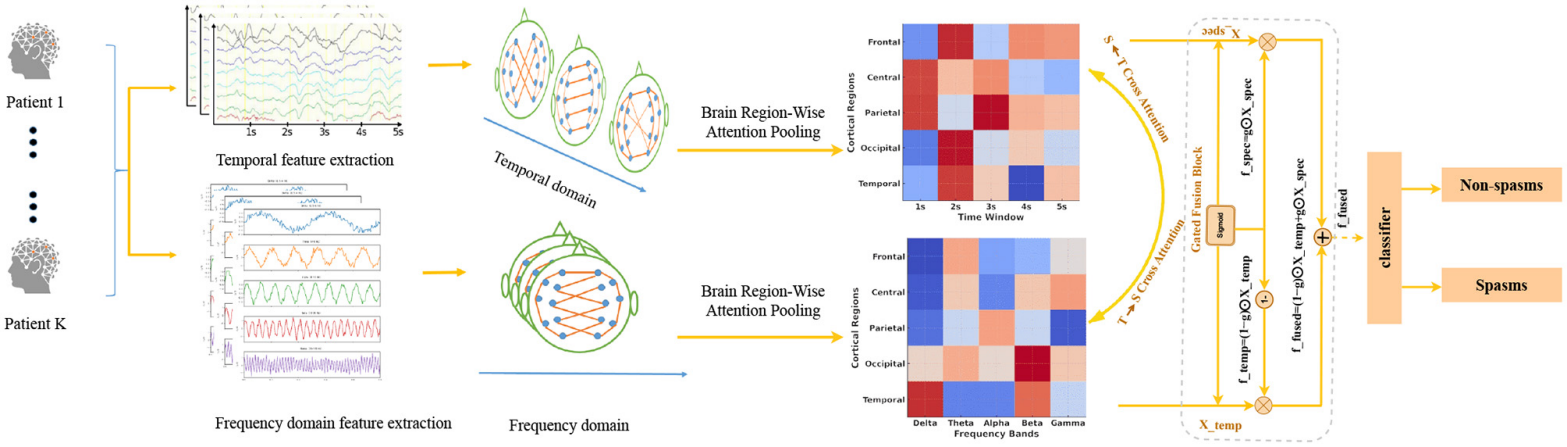
The overall architecture of CMTS-GNN. The CMTS-GNN model is designed to classify EEG segments as spasm or non-spasm events by leveraging both temporal and spectral characteristics of EEG data. Raw EEG signals are processed in parallel through temporal and spectral branches. Within each branch, attention-based pooling aggregates features across anatomically grouped brain regions, generating region-wise temporal and spectral feature maps. A bidirectional cross-modal attention module is then applied to enable effective interaction between temporal and spectral representations, enhancing the features based on the complementary information from both modalities. Subsequently, the attention-refined features are adaptively integrated with the original representations through gated fusion blocks, where learnable sigmoid gates dynamically control the contribution of each modality. The resulting fused representation encodes rich and complementary spatiotemporal information, which is ultimately fed into a classifier for final decision-making between non-spasm and spasm events.

Infantile spasm EEG signals are characterized by substantial heterogeneity and rapidly shifting spatiotemporal patterns, making them difficult to model with conventional sequence-based approaches. Such models often struggle to capture the non-Euclidean topology inherent to EEG channel arrangements while also integrating complementary cues from temporal and spectral domains. To overcome these challenges, the proposed CMTS-GNN unifies three key operations into a single pipeline: it first extracts temporal features at multiple scales, then performs edge-aware graph reasoning enriched with brain-region-wise pooling, and finally applies bidirectional cross-modal attention coupled with gated fusion. The resulting architecture simultaneously models waveform dynamics, frequency rhythms, and inter-channel connectivity, delivering a cohesive and clinically relevant framework for EEG analysis. The network architecture of CMTS-GNN is shown in Figure 1.

#### 2.5.1 Multi-scale temporal feature extraction

Given a time-domain EEG segment *X*^(*t*)^ ∈ ℝ^*N*×*T*^ with *N* channels and *T* samples per channel, three parallel 1-D convolution branches with kernel sizes {100, 50, 25} are applied to capture long-range, medium-range, and short-range temporal dependencies. Each branch consists of a convolution layer, batch normalization, and ReLU activation:


(10) $$\begin{array}{c} H_{k}=\text{ReLU}(\text{BN}\left(\text{Conv}1{\text{D}}_{k}\left(X^{\left(t\right)}\right)\right)),\;k\in \left\{100,50,25\right\}. \end{array}$$

Global average pooling over the temporal dimension produces compact channel-wise descriptors. If the sequence length after convolution is *L*_*k*_:


(11) $$\begin{array}{c} {\bar{H}}_{k}\left(i,:\right)=\frac{1}{L_{k}}\overset{L_{k}}{\underset{t=1}{\sum }}H_{k}\left(i,t,:\right),\;i=1,\ldots ,N. \end{array}$$

The pooled features from all branches are concatenated and linearly transformed into a shared hidden space of width *D*:


(12) $$\begin{array}{c} H_{\text{ms}}^{\left(t\right)}=\phi \left(\left[{\bar{H}}_{100}\;∥\;{\bar{H}}_{50}\;∥\;{\bar{H}}_{25}\right]\right),\;\phi \left(\cdot \right)=W_{f}\left(\cdot \right)+b_{f}, \end{array}$$

where $H_{\text{ms}}^{\left(t\right)}\in {\mathbb{R}}^{N\times D}$. This step generates scale-robust temporal embeddings that retain both transient bursts and contextual information.

#### 2.5.2 Edge-aware graph encoding and brain-region pooling

In both temporal and spectral streams, EEG channels are modeled as graph nodes, with edges encoding functional connectivity derived from DFC or wPLI. Node features are projected to a common width *D*:


(13) $$\begin{array}{c} {\hat{X}}^{\left(t\right)}=W_{p}^{\left(t\right)}H_{\text{ms}}^{\left(t\right)}+b_{p}^{\left(t\right)},\;{\hat{X}}^{\left(s\right)}=W_{p}^{\left(s\right)}X_{\text{in}}^{\left(s\right)}+b_{p}^{\left(s\right)}. \end{array}$$

For an edge (*i, j*) with scalar attribute $a_{ij}^{\left(m\right)}$ in modality *m* ∈ {*t, s*}, an MLP outputs an edge-specific kernel:


(14) $$\begin{array}{c} W_{ij}^{\left(m\right)}=\text{reshape}\left(\text{ML}{\text{P}}^{\left(m\right)}\left(a_{ij}^{\left(m\right)}\right)\right)\in {\mathbb{R}}^{D\times D}. \end{array}$$

Node features are updated via mean aggregation over neighbors, followed by ReLU and LayerNorm:


(15) $$\begin{array}{c} Z_{i}^{\left(m\right)}=\text{LN}\left(\text{ReLU}\left(\frac{1}{\left|N\left(i\right)\right|}\underset{j\in N\left(i\right)}{\sum }W_{ij}^{\left(m\right)}{\hat{X}}_{j}^{\left(m\right)}\right)\right). \end{array}$$

Channels are grouped into five anatomical regions (frontal, central, parietal, occipital, temporal) based on the 10-20 system. Within each region *r*, attention pooling computes:


(16) $$\begin{array}{c} s_{i}^{\left(m,r\right)}={\left(w_{r}^{\left(m\right)}\right)}^{⊤}Z_{i}^{\left(m\right)},\;\alpha_{i}^{\left(m,r\right)}=\frac{e^{s_{i}^{\left(m,r\right)}}}{\sum_{j\in R_{r}}e^{s_{j}^{\left(m,r\right)}}},\\ u_{r}^{\left(m\right)}=\sum_{i\in R_{r}}\alpha_{i}^{\left(m,r\right)}Z_{i}^{\left(m\right)}. \end{array}$$

Stacking *R* = 5 regions yields *U*^(*m*)^ ∈ ℝ^*R*×*D*^.

#### 2.5.3 Cross-modal interaction and gated fusion

At the region level, temporal and spectral matrices interact via bidirectional multi-head cross-attention. For the temporal to spectral direction:


(17) $$\begin{array}{c} {Ũ}^{\left(t\right)}=\left[\text{Conca}{\text{t}}_{h=1}^{H}\text{ Softmax}\left(\frac{Q^{\left(h\right)}K^{\left(h\right)⊤}}{\sqrt{d_{h}}}\right)V^{\left(h\right)}\right]W_{O}, \end{array}$$

with $Q^{\left(h\right)}=U^{\left(t\right)}W_{Q}^{\left(h\right)},K^{\left(h\right)}=U^{\left(s\right)}W_{K}^{\left(h\right)},V^{\left(h\right)}=U^{\left(s\right)}W_{V}^{\left(h\right)}$. The spectral → time direction is analogous.

Gated fusion adaptively combines original and enhanced features:


(18) $$\begin{array}{c} {û}_{r}^{\left(t\right)}=g_{t}^{\left(r\right)}⊙{ũ}_{r}^{\left(t\right)}+\left(1-g_{t}^{\left(r\right)}\right)⊙u_{r}^{\left(t\right)}, \end{array}$$

(19) $$\begin{array}{c} {û}_{r}^{\left(s\right)}=g_{s}^{\left(r\right)}⊙{ũ}_{r}^{\left(s\right)}+\left(1-g_{s}^{\left(r\right)}\right)⊙u_{r}^{\left(s\right)}, \end{array}$$

where $g_{t}^{\left(r\right)}$ and $g_{s}^{\left(r\right)}$ are sigmoid gates from concatenated inputs.

Fused features from all regions are concatenated, flattened, and passed to a two-layer fully connected classifier with dropout:


(20) $$\begin{array}{c} y=W_{2}\text{ Dropout }\left(\text{ReLU}\left(W_{1}f+b_{1}\right)\right)+b_{2},\;\hat{p}=\sigma \left(y\right). \end{array}$$

This sequential design–multi-scale temporal encoding, graph reasoning with anatomical priors, cross-modal alignment, and gated fusion–produces robust, interpretable segment-level predictions.

## 3 Experiments and results

### 3.1 Experimental environment

Our method is implemented using PyTorch and trained on an Ubuntu server equipped with an Intel^®^ Xeon^®^ Gold 6133 @ 2.50GHz CPU and an NVIDIA 3090Ti GPU. The Adam optimizer is adopted for training, with the learning rate set to 0.01. The entire network is trained with a batch size of 32 for a total of 150 epochs. Due to sample imbalance, a focal loss function is used as the loss criterion, which is proposed by Lin et al. (29).

### 3.2 Evaluation metrics


(21) $$\begin{array}{c} \text{Accuracy}=\frac{TP+TN}{TP+TN+FP+FN} \end{array}$$

(22) $$\begin{array}{c} \text{Recall}=\frac{TP}{TP+FN} \end{array}$$

(23) $$\begin{array}{c} \text{Precision}=\frac{TP}{TP+FP} \end{array}$$

(24) $$\begin{array}{c} \text{Specificity}=\frac{TN}{TN+FP} \end{array}$$

(25) $$\begin{array}{c} \text{AUC}=\frac{\underset{i\in P}{\sum }r_{i}-\frac{P\left(P+1\right)}{2}}{PN},\;r_{i}=\mathrm{rank}\left(s_{i}\right), \end{array}$$

Here, *TP*, *TN*, *FP*, and *FN* represent *True Positive, True Negative, False Positive*, and *False Negative*, respectively.

### 3.3 Comparative experiment

**Figure 2:**
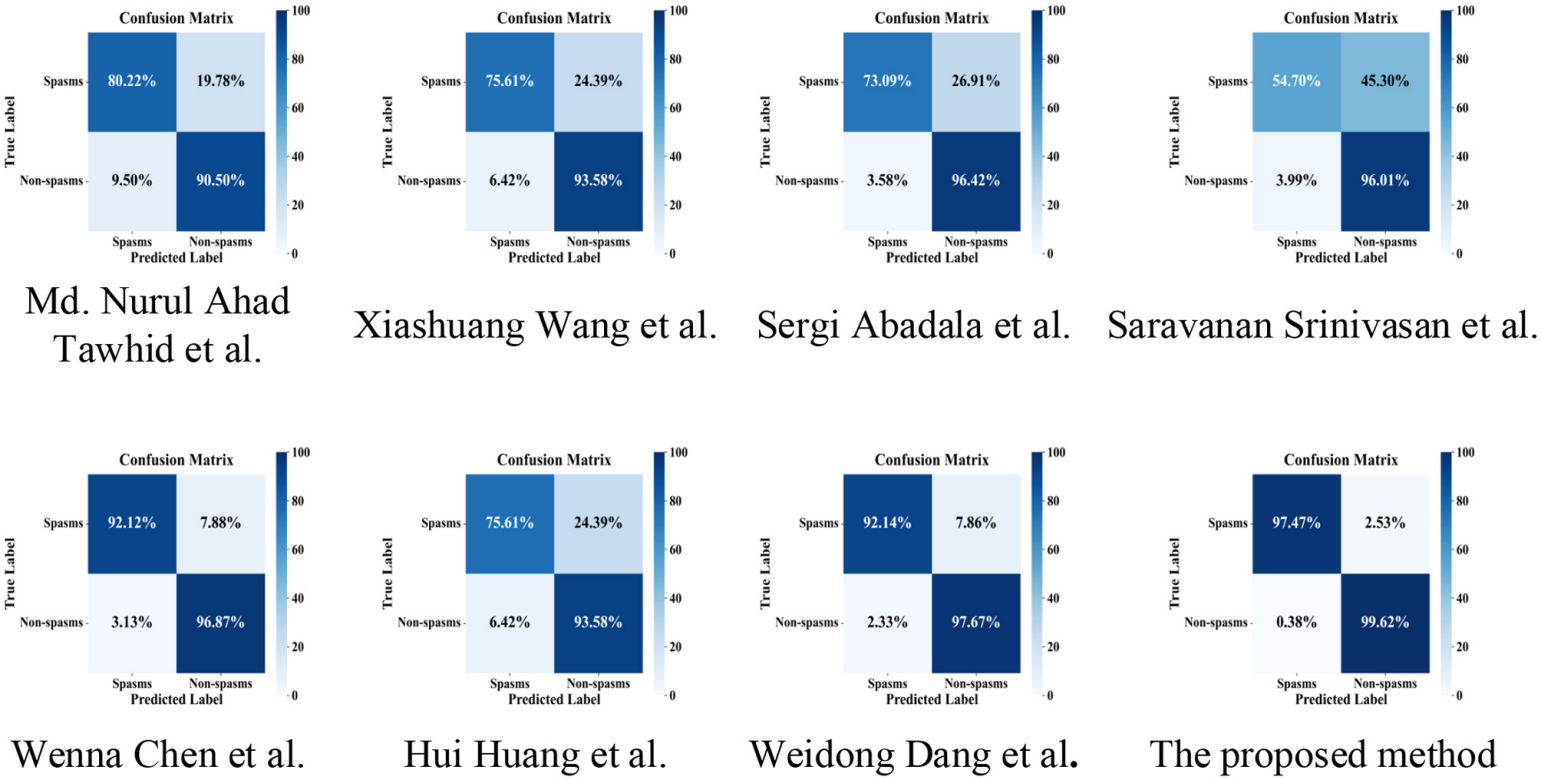
Comparison of confusion matrices between the proposed method and state-of-the-art methods on dataset A. The colorbar indicates the percentage, which is row-normalized.

To provide a comprehensive evaluation of our proposed CMTS-GNN model, we reproduced several representative state-of-the-art methods and conducted a unified performance comparison on Dataset A using 5-fold cross-validation. While the official implementations of some models were not publicly available, we carefully replicated the architectures and training procedures based on the original papers to ensure high fidelity. The experimental results are summarized in Table 2 and further illustrated through the confusion matrices (Figure 2).

**Table 2:** Performance comparison between the proposed method and state-of-the-art methods using 5-fold cross-validation on dataset A.

**Author**	**Accuracy (%)**	**Pre (%)**	**Recall (%)**	**F1 (%)**	**AUC (%)**
Md. Nurul Ahad Tawhid et al. (30)	87.84	75.89	80.22	77.86	89.63
*δ (SD)*	±1.25	±2.10	±2.35	±2.05	±1.50
Xiashuang Wang et al. (31)	88.78	81.44	75.61	78.18	93.38
*δ (SD)*	±1.10	±1.50	±2.20	±1.65	±0.90
Sergi Abadala et al. (32)	90.21	88.38	73.09	79.96	93.75
*δ (SD)*	±0.95	±1.20	±2.10	±1.50	±0.85
Saravanan Srinivasan et al. (33)	84.90	83.63	54.70	66.07	88.10
*δ (SD)*	±1.80	±1.70	±3.50	±2.90	±1.40
Wenna Chen et al. (34)	95.55	91.65	92.12	91.74	99.13
*δ (SD)*	±0.80	±1.00	±0.90	±0.95	±0.30
Hui Huang et al. (35)	90.09	83.57	78.64	81.03	86.47
*δ (SD)*	±0.90	±1.20	±1.50	±1.20	±1.00
Weidong Dang et al. (36)	96.12	93.65	92.14	92.77	94.87
*δ (SD)*	±0.70	±0.90	±1.00	±0.85	±0.80
The proposed method	99.02	98.96	97.47	98.20	99.27
*δ (SD)*	±0.35	±0.40	±0.55	±0.45	±0.25

δ (SD) reports the standard deviation across the five folds for each metric.

Hybrid architectures such as the ConvLSTM-based model proposed by Md. Nurul Ahad Tawhid et al. (30) and the CNN-LSTM framework by Xiashuang Wang et al. (31) combine convolutional and recurrent layers to capture spatiotemporal dependencies in EEG signals. When evaluated on our dataset, the ConvLSTM model achieved an accuracy of 87.84% and a recall of 80.22%, but showed limited precision at 75.89%, resulting in an F1-score of 77.86% and an AUC of 89.63%. CNN-LSTM improved the overall accuracy to 88.78% and precision to 81.44%, though its recall declined to 75.61%, indicating reduced sensitivity to spasm events. The confusion matrices for both models reveal a noticeable presence of off-diagonal elements, reflecting misclassifications likely caused by the domain shift from adult to infant EEG. Sergi Abadala et al. (32) proposed a Graph Transformer Network (GTN) designed to model inter-channel dependencies in EEG data. Although it achieved a precision of 88.38% in our experiments, the recall was only 73.09%, suggesting under-detection of spasm episodes. Likewise, the hybrid 3D-Denoising Convolutional Autoencoder (3D-DCAE) + Bi-LSTM model by Srinivasan et al. (33) exhibited the weakest performance among all compared models, with a recall of just 54.70% and an F1-score of 66.07%, indicating limited generalizability to infantile EEG patterns.

Models incorporating attention mechanisms and multi-level feature fusion have shown relatively better adaptability to our dataset. The 1D-CNN with attention-based feature fusion, proposed by Wenna Chen et al. (34), achieved strong performance, with 95.55% accuracy, 91.65% precision, 92.12% recall, and an F1-score of 91.74%, and its confusion matrix showed minimal off-diagonal misclassifications. Similarly, the multiband 3D-CNN with attention mechanisms by Hui Huang et al. (35) yielded competitive performance. The Multi-branch Deep Convolutional Neural Network (MDCNN) proposed by Weidong Dang et al. (36) achieved 96.12% accuracy, 93.65% precision, 92.14% recall, and an F1-score of 92.77%, highlighting the advantages of deeper convolutional structures in capturing EEG dynamics.

In comparison, the proposed CMTS-GNN model achieved state-of-the-art results, with an accuracy of 99.02%, precision of 98.96%, recall of 97.47%, F1-score of 98.20%, and AUC of 99.27%. CMTS-GNN encloses the largest area across all metrics, signifying superior balance between sensitivity and specificity. Moreover, the confusion matrix of CMTS-GNN shows near-perfect classification, with negligible false positives and false negatives, in contrast to the scattered misclassifications observed in other methods.

These results demonstrate that the integration of multiscale temporal encoding, edge-aware graph modeling, cross-modal attention, and brain region-wise pooling enables CMTS-GNN to effectively capture complex spatiotemporal-frequency dependencies in EEG data. Consequently, our method not only surpasses existing approaches in classification performance but also sets a new benchmark in achieving a balanced and reliable detection of infant spasms.

### 3.4 Ablation experiments

To verify the contribution of each module in our model, we designed several variant models. First, we use cross-modal fusion between temporal and frequency domains as the baseline model, and then progressively integrate additional modules to form the complete model. The specific configurations are as follows:

- **Variant A (Cross-modal fusion):** We use a network that performs cross-modal fusion between temporal-domain and frequency-domain graphs as the baseline model.
- **Variant B (+** **Multi-Head Attention):** Based on the cross-modal fusion, we add a multi-head attention mechanism.
- **Variant C (+** **Brain Region-Wise Attention Pooling):** We enhance the cross-modal fusion model by introducing Brain Region-Wise Attention Pooling.
- **Variant D (+** **Multi-Head Attention** **+** **Brain Region-Wise Attention Pooling):** We incorporate both Multi-Head Attention and Brain Region-Wise Attention Pooling into the cross-modal fusion framework (our proposed model CMTS-GNN).

To evaluate the contribution of each component in the proposed CMTS-GNN model, we conducted a comprehensive ablation study by designing four variant models with progressive integration of core modules. As shown in Table 3, the baseline model utilizing only cross-modal fusion between temporal and spectral features (Variant A) yielded the lowest performance across all metrics, with an accuracy of 76.86% and F1-score of 52.65%. Introducing the multi-head attention mechanism (Variant B) significantly enhanced performance, boosting the F1-score to 89.72%, highlighting its effectiveness in modeling inter-modal dependencies. Further incorporating Brain Region-Wise Attention Pooling (Variant C) led to substantial improvements across all evaluation metrics, with a notable increase in precision (96.62%) and specificity (98.78%), indicating the benefit of anatomical priors in feature aggregation. Finally, the full model (Variant D), integrating both multi-head attention and brain-region-wise pooling, achieved the highest performance with an accuracy of 99.02%, F1-score of 98.20%, and specificity of 99.05%. These results demonstrate that each module contributes incrementally and synergistically to the overall performance, validating the design of the CMTS-GNN architecture.

**Table 3:** The comparison of experimental results from ablation experiments.

**Model**	**Accuracy (%)**	**Pre (%)**	**Recall (%)**	**F1 (%)**	**Specificity (%)**
Cross-modal fusion	76.86	58.66	47.99	52.65	87.48
*δ (SD)*	±2.10	±2.40	±3.20	±2.80	±1.90
Cross-modal fusion + Multi-head attention	94.37	87.83	91.88	89.72	95.32
*δ (SD)*	±0.95	±1.10	±1.25	±1.05	±0.90
Cross-modal + Brain region-wise attention pooling	97.76	96.62	95.01	95.83	98.78
*δ (SD)*	±0.70	±0.85	±1.00	±0.90	±0.60
The proposed method	99.02	98.96	97.47	98.20	99.05
*δ (SD)*	±0.35	±0.40	±0.55	±0.45	±0.40

δ (SD) reports the standard deviation across the ablation experiments for each metric.

### 3.5 Leave-one-patient-out cross-validation on dataset A

To rigorously evaluate the generalizability of the proposed CMTS-GNN model across different subjects, we conducted a Leave-One-Patient-Out Cross-Validation (LOPO-CV) experiment. In this setting, the dataset comprising 40 infant patients was partitioned such that, in each iteration, the EEG recordings from one patient were held out as the test set, while the remaining 39 patients' data were used for training. This process was repeated 40 times, ensuring that each patient served exactly once as the test subject. LOPO-CV offers a stringent and subject-independent evaluation protocol, particularly suitable for medical applications where inter-subject variability is high. It allows us to assess the model's robustness and its ability to generalize to previously unseen patients, a critical requirement for real-world clinical deployment in infantile spasm detection. Because Table 4 shows substantial and heterogeneous class imbalance at the subject level, we explicitly balanced our cross-validation splits. For 5-fold CV, we used a grouped, stratified split at the patient level: patients were ordered by their spasm counts and assigned to folds in a round-robin manner so that each fold approximated the global spasm/non-spasm ratio and contained comparable EEG hours; no re-sampling was applied on the validation fold.

**Table 4:** Performance of CMTS-GNN using leave-one-patient-out cross-validation on dataset A.

**Number**	**Accuracy (%)**	**Pre (%)**	**Recall (%)**	**F1 (%)**	**Specificity (%)**
01	93.93	87.49	87.49	87.49	95.99
02	88.46	71.43	83.30	76.92	90.00
03	100.00	100.00	100.00	100.00	100.00
04	91.55	82.61	90.48	86.36	92.00
05	100.00	100.00	100.00	100.00	100.00
06	97.49	90.91	100.00	95.24	96.67
07	94.23	88.89	94.12	91.39	94.29
08	91.30	72.73	88.89	79.68	91.89
09	92.86	82.35	93.33	87.45	92.68
10	100.00	100.00	100.00	100.00	100.00
11	92.00	75.00	90.00	81.82	92.50
12	90.74	78.57	84.62	81.25	92.68
13	92.68	85.71	92.31	88.89	92.86
14	89.74	72.73	88.89	80.00	90.00
15	89.36	75.00	81.82	78.26	91.67
16	100.00	100.00	100.00	100.00	100.00
17	100.00	100.00	100.00	100.00	100.00
18	89.23	73.33	78.57	75.81	92.16
19	98.46	94.44	100.00	97.14	97.92
20	94.00	85.71	92.31	88.83	94.59
21	95.54	90.91	93.75	92.31	96.25
22	92.68	77.78	87.50	82.35	93.94
23	100.00	100.00	100.00	100.00	100.00
24	98.11	100.00	92.31	95.99	100.00
25	97.14	100.00	91.30	95.45	100.00
26	94.00	84.62	91.67	88.00	94.74
27	95.38	90.00	94.74	92.28	95.65
28	92.50	80.00	88.89	84.21	93.55
29	93.67	91.30	87.50	89.32	96.36
30	100.00	100.00	100.00	100.00	100.00
31	90.91	89.47	77.27	83.02	96.36
32	100.00	100.00	100.00	100.00	100.00
33	90.38	76.92	83.33	79.96	92.50
34	89.39	73.68	87.50	79.69	90.00
35	100.00	100.00	100.00	100.00	100.00
36	97.40	94.12	94.12	94.12	98.33
37	91.14	76.19	88.89	82.05	91.80
38	90.98	100.00	73.81	84.93	100.00
39	99.14	100.00	97.29	98.63	100.00
40	94.31	87.88	90.63	89.27	96.67

The leave-one-patient-out cross-validation results, as presented in Table 4, demonstrate the strong generalization and robustness of the CMTS-GNN model for infantile spasm detection across a diverse cohort of 40 subjects. Notably, 10 patients, such as numbers 3, 5, 10, 16, 17, 23, 30, 32, 35, and 39, exhibited perfect scores for all metrics, reflecting cases where the model could fully separate spasm from non-spasm events. The majority of samples were correctly classified, indicating both high sensitivity and specificity. Given the pronounced class imbalance, accuracy alone can be inflated, therefore we interpret performance in light of this balance and emphasize precision, recall, specificity, F1-score, accuracy so that each subject contributes equally. In subjects with very few spasms, precision is expected to be lower because non-spasm segments dominate, whereas consistently high recall indicates that true spasm episodes are still captured despite imbalance. False negatives and false positives were relatively rare, but some patients–such as number 2 and number 18,displayed lower precision, resulting in more false positive predictions. For example, in these instances, the confusion matrix showed an increased number of non-spasm samples misclassified as spasms, suggesting that patient-specific signal variability or noise may present challenges for the model. Despite this, recall remained above 75 percent for nearly all patients, underscoring the model's robustness in capturing true spasm episodes even in less distinct or noisy EEG segments. The overall distribution of LOPO-CV metrics reveals a low standard deviation, reflecting consistent model performance and minimal overfitting to individual subjects. Furthermore, the confusion matrix did not reveal any subject with systematic misclassification of either spasms or non-spasms, supporting the patient-independence and clinical reliability of CMTS-GNN. These results validate that CMTS-GNN can effectively generalize across patients and holds significant potential for real-world deployment in clinical settings for automated infantile spasm detection. The overall distribution of LOPO-CV metrics reveals a low standard deviation, reflecting consistent model performance and minimal overfitting to individual subjects. Furthermore, the confusion matrix did not reveal any subject with systematic misclassification of either spasms or non-spasms, supporting the patient-independence and clinical reliability of CMTS-GNN. These results validate that CMTS-GNN can effectively generalize across patients and holds significant potential for real-world deployment in clinical settings for automated infantile spasm detection.

### 3.6 Explainability of model decisions

To provide insight into the decision-making process of our deep learning model, we employed the gradient multiplied by input attribution method. This approach, originally described by Karen Simonyan et al. (37) in 2013 in the context of saliency maps, quantifies feature importance by computing the element-wise product of the input and the gradient of the output with respect to that input. This method has since been widely adopted in the field of neural network interpretability, and was further developed by Mukund Sundararajan et al. (38) in 2017 through the introduction of Integrated Gradients. The resulting relevance scores reflect the direct contribution of each input feature to the model's prediction, offering an intuitive and computationally efficient means of interpreting complex models. In the context of electroencephalogram (EEG) analysis, the application of gradient multiplied by input attribution is particularly important (16, 37, 39). EEG signals are high-dimensional and spatially distributed, with substantial variability across both subjects and brain regions. Traditional deep learning models, while powerful in capturing nonlinear spatiotemporal dependencies, often lack transparency, making it difficult to assess which channels or temporal segments drive the network's predictions. By employing gradient multiplied by input attribution, we can generate channel-wise or region-wise relevance maps, enabling neuroscientific interpretation and clinical validation of model behavior. This not only enhances trust in automated EEG classification systems, but also helps uncover physiologically meaningful patterns that may underlie epileptic activity or other neurological events.

Given that the proposed cross-modal fusion architecture is capable of simultaneously integrating temporal and spectral graph features, we further designed a weighted fusion mechanism for the attribution scores, combining the channel contributions from both modalities in a weighted manner. The fusion coefficient was set to 0.5 to ensure equal representation of temporal and spectral information. Specifically, we applied the gradient multiplied by input method to compute the attribution scores for each channel in both the temporal and spectral domains, and then aggregated these scores using the weighted scheme to obtain the final channel relevance scores. To facilitate spatial pattern comparison across different samples, we averaged the channel scores along the temporal dimension for each sample to obtain a single spatial vector. Finally, based on the international standard 10–20 electrode system, we visualized the model's decision basis by plotting EEG topographic maps.

**Figure 3:**
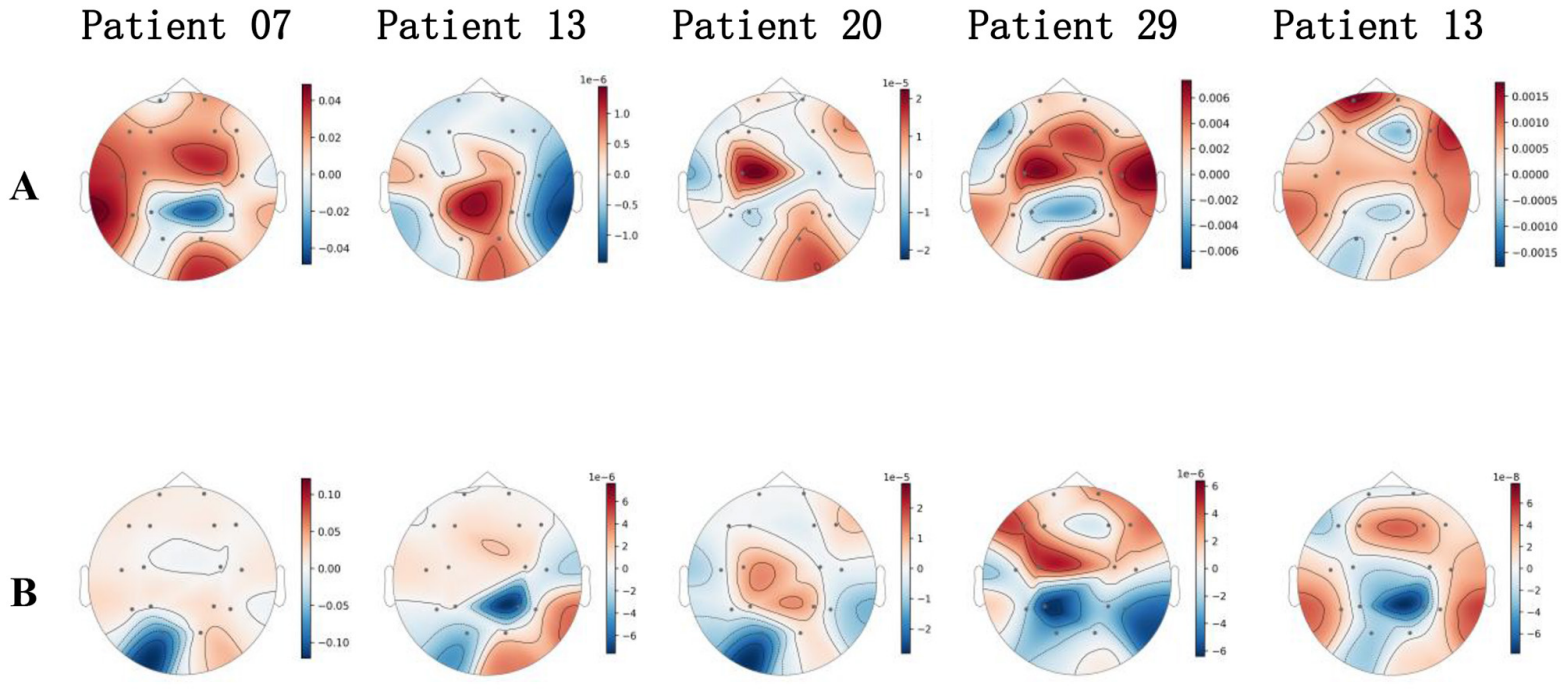
Topographic maps of attribution scores are shown for samples from five infants with spasms. Higher normalized attribution scores indicate features that are more relevant for the model's classification decision, whereas lower scores represent less relevant or irrelevant inputs. **(A)** displays attribution maps for spasm samples, while **(B)** corresponds to non-spasm samples.

As shown in Figure 3, by visualizing the topographic maps of attribution scores for several infant spasm samples, it can be observed that when the model identifies spasm events, it notably focuses on neural activity in the frontal, central, and temporal regions. These areas consistently display higher positive attribution scores in most spasm samples, indicating their critical discriminative value in the model's classification decisions. In contrast, channels in the occipital region tend to exhibit negative or low contributions, suggesting that this region is not important for spasm recognition. The spatial activation pattern remains highly consistent across different samples, and also demonstrates individualized lateralization of epileptogenic zones, reflecting the model's sensitivity to the potential distribution of epileptic foci. Importantly, these attribution results are highly consistent with findings from clinical EEG research, which indicate that infantile spasms most frequently originate from the frontal lobe, central motor cortex, and temporal pole regions, as documented by Lux et al. (40) and Watanabe et al. (41). This correspondence confirms the neurophysiological validity and medical relevance of the model's interpretability, further supporting the value of deep models in spasm prediction.

In contrast, analysis of the attribution topographic maps for non-spasm samples shows that when the model identifies non-spasm states, the overall distribution of channel relevance scores becomes more diffuse, with no concentrated activation regions. Most channels present attribution scores close to zero or mildly negative, especially in the occipital and central areas, which consistently show a suppressive contribution in multiple samples. This suggests that the model derives non-spasm evidence from these regions. Compared to the prominent frontal and temporal activation observed in spasm samples, the spatial discriminability and activation magnitude in non-spasm samples are substantially reduced. This trend demonstrates that the model can effectively distinguish spatial features under different clinical states, providing visual evidence for its stability and reliability in practical clinical applications.

From a cognitive network perspective, CMTS-GNN yields explanations at the level of large-scale functional systems rather than isolated channels. The brain-region-wise attention pooling in Equation (16) produces region embeddings that serve as proxies for canonical systems. Bidirectional cross-modal attention together with the gated fusion in Equations 18–19 then quantifies how evidential support flows between these systems across temporal and spectral representations. Aggregating gradient × input attributions within each anatomically defined region provides a decomposable “network evidence” profile per segment, revealing that spasm decisions are primarily driven by fronto-central and anterior temporal systems, with consistent suppression or low evidence in occipital cortex. This network-level pattern accords with circuits subserving early sensorimotor control, cognitive control, and affective reactivity, and thus offers a cognitively meaningful account of why the model classifies a segment as spasm vs. non-spasm. Practically, per-region evidence can be surfaced alongside predictions to support clinical review and to track patient-specific lateralization over time, linking model outputs to interpretable cognitive networks and facilitating biomarker development for downstream mental-health modeling.

### 3.7 Leave-one-patient-out cross-validation on dataset B

To further evaluate the generalization capability of the proposed CMTS-GNN model across different epilepsy types and EEG backgrounds, we conducted transfer testing on the public CHB-MIT epilepsy dataset. The CHB-MIT dataset consists of long-term EEG recordings from multiple epilepsy patients, encompassing a wide spectrum of seizure types and exhibiting background activity and ictal patterns that differ substantially from those observed in infantile spasms. Employing this dataset as an independent test set not only imposes stricter requirements on model robustness and cross-domain adaptability, but also more accurately simulates real-world clinical scenarios. For data preprocessing, all EEG recordings–both seizure and non-seizure segments–were uniformly segmented into five-second epochs to standardize input length and enhance temporal resolution for model analysis. In addition, to ensure consistency across samples and facilitate robust cross-subject evaluation, we retained only the 18 EEG channels that were common to all recordings: FP2-F4, C4-P4, T8-P8, F7-T7, FP1-F3, FP1-F7, P7-O1, F4-C4, T7-P7, P8-O2, P3-O1, F8-T8, FZ-CZ, FP2-F8, CZ-PZ, F3-C3, C3-P3 and P4-O2.

To avoid class imbalance and to provide a fair evaluation of the model's discriminative ability, we adopted a balanced scheme with equal proportions of positive and negative samples. Several representative and state-of-the-art methods were selected for unified performance comparison on Dataset B using five-fold cross-validation. In addition, we employed a leave-one-subject-out (LOSO) cross-validation strategy, where the EEG data of each patient was sequentially used as the test set, while the data from the remaining patients served as the training set. This approach provides a comprehensive assessment of the model's generalization ability and robustness across different individuals. Detailed experimental results are presented in Tables 5, 6.

**Table 5:** Performance comparison between the proposed method and state-of-the-art methods using 5-fold cross-validation on dataset B.

**Author**	**Accuracy (%)**	**Pre (%)**	**Recall (%)**	**F1 (%)**	**AUC (%)**
Md. Nurul Ahad Tawhid et al. (30)	98.27	97.52	98.96	98.23	98.69
Xiashuang Wang et al. (31)	97.92	97.71	98.04	97.88	98.15
Wenna Chen et al. (34)	98.30	97.98	98.56	98.27	99.04
The proposed method	98.54	98.31	98.71	98.47	98.87

**Table 6:** Performance of CMTS-GNN using leave-one-patient-out cross-validation on dataset B.

**Number**	**Accuracy (%)**	**Pre (%)**	**Recall (%)**	**F1 (%)**	**Specificity (%)**
01	98.31	98.83	97.70	98.27	98.89
02	89.66	86.67	92.86	89.66	86.67
03	100.00	100.00	100.00	100.00	100.00
04	81.91	88.57	70.45	78.48	91.99
05	100.00	100.00	100.00	100.00	100.00
06	91.80	83.87	100.00	91.15	85.71
07	93.88	95.24	90.91	93.02	96.30
08	89.82	87.29	92.79	89.94	86.96
09	96.17	96.42	97.25	96.81	94.46
10	100.00	100.00	100.00	100.00	100.00
11	96.26	96.63	95.56	96.09	96.91
12	93.42	91.66	94.74	93.16	92.22
13	85.16	87.14	81.33	84.14	88.75
14	87.88	100.00	74.19	85.19	100.00
15	94.64	94.78	90.83	92.71	96.95
16	83.33	74.99	85.71	79.99	81.82
17	89.33	96.43	79.41	87.01	97.56
18	89.36	92.31	83.72	87.74	94.12
19	88.24	100.00	71.43	83.33	100.00
20	88.79	83.33	96.15	89.29	81.82
21	87.72	95.00	76.00	84.33	96.88
22	98.39	100.00	96.55	98.25	100.00
23	96.82	97.33	96.05	96.62	97.53

All values are percentages (%). **Pre** denotes precision.

**Figure 4:**
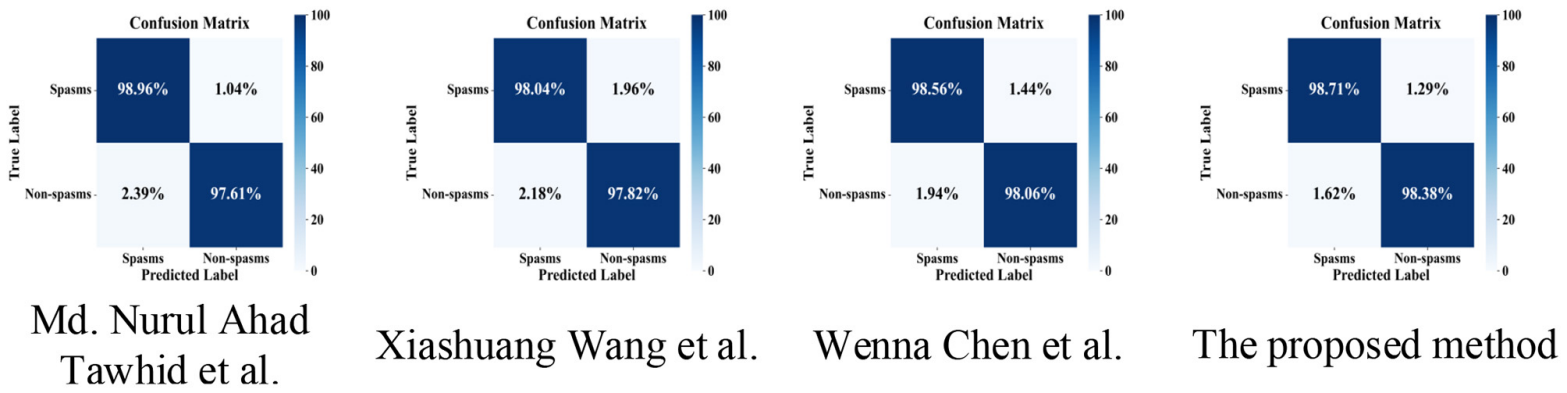
Comparison of confusion matrices between the proposed method and state-of-the-art methods on dataset A. The colorbar indicates the percentage, which is row-normalized.

The proposed method demonstrated outstanding overall performance in the five-fold cross-validation experiments conducted on the CHB-MIT public dataset. Specifically, this method outperformed other comparative approaches in all evaluation metrics, including accuracy (98.54%), precision (98.31%), recall (98.71%), and F1-score (98.47%). In comparison, the method by Wenna Chen et al. achieved the highest AUC (99.04%), but its other metrics—such as accuracy and F1-score–were slightly lower than those of the proposed method. The related metrics of Tawhid et al. (30) and Wang (31) were all inferior to those of our method, with particularly noticeable gaps in recall and precision. As shown in Figure 4, the confusion matrix provides an intuitive reflection of the classification performance on both positive and negative samples. It can be observed that the proposed method achieves higher true positive rate (98.71%) and true negative rate (98.38%) than the comparative methods, indicating fewer missed detections and false alarms in practical detection. While other methods also perform well, some exhibit higher error rates in negative sample discrimination; for example, the true negative rate of the Tawhid et al. (30) method is 97.61%, slightly lower than that of the proposed method. In summary, the proposed method consistently outperforms several state-of-the-art algorithms in the five-fold cross-validation experiments on the CHB-MIT public dataset. Not only does it achieve optimal results in accuracy, precision, recall, and F1-score, but its AUC value is also close to the highest, indicating strong potential for application in the automatic detection of infantile spasms.

## 4 Conclusion

We proposed CMTS-GNN, a cross-modal temporal-spectral graph neural network for automated infantile spasm detection from EEG, and demonstrated state-of-the-art performance with strong generalizability and interpretability. On the dedicated infant spasm dataset, CMTS-GNN reached 99.02% accuracy, 98.96% precision, 97.47% recall, 98.20% F1, and 99.27% AUC under five-fold evaluation, and exhibited robust patient-independent generalization in leave-one-patient-out testing with multiple subjects achieving perfect scores. Cross-domain transfer to CHB-MIT confirmed robustness under distribution shift, yielding 98.54% accuracy, 98.31% precision, 98.71% recall, 98.47% F1, and 98.87% AUC in five-fold evaluation, while most patients surpassed 90% accuracy in leave-one-subject-out testing. Attribution analysis highlighted frontal, central, and temporal regions during spasm detections in line with clinical knowledge. These results establish CMTS-GNN as an accurate, generalizable, and clinically interpretable solution for infantile spasm detection and motivate future work on larger and more diverse cohorts, integration of additional physiological signals, and refined interpretability to support clinical deployment.

## Data Availability

The raw data supporting the conclusions of this article will be made available by the authors, without undue reservation.
